# Potential radiation dose reduction in clinical photon-counting CT by the small pixel effect: ultra-high resolution (UHR) acquisitions reconstructed to standard resolution

**DOI:** 10.1007/s00330-023-10499-1

**Published:** 2023-12-22

**Authors:** Markel Fix Martinez, Laura Klein, Joscha Maier, Lukas Thomas Rotkopf, Heinz-Peter Schlemmer, Stefan Oswald Schönberg, Marc Kachelrieß, Stefan Sawall

**Affiliations:** 1 Division of X-Ray Imaging and CT, German Cancer Research Center (DKFZ), Im Neuenheimer Feld 280, 69120 Heidelberg, Germany; 2 Division of Radiology, German Cancer Research Center (DKFZ), Im Neuenheimer Feld 280, 69120 Heidelberg, Germany; 3 Department of Clinical Radiology and Nuclear Medicine, University Hospital Mannheim, Theodor-Kurz-Ufer 1-3, 68167 Mannheim, Germany; 4 Medical Faculty, Heidelberg University, Im Neuenheimer Feld 672, 69120 Heidelberg, Germany

**Keywords:** Computed tomography (X-ray), Dosage (Radiation), Phantom (Imaging)

## Abstract

**Objective:**

To assess the potential dose reduction achievable with clinical photon-counting CT (PCCT) in ultra-high resolution (UHR) mode compared to acquisitions using the standard resolution detector mode (Std).

**Materials and methods:**

With smaller detector pixels, PCCT achieves far higher spatial resolution than energy-integrating (EI) CT systems. The reconstruction of UHR acquisitions to the lower spatial resolution of conventional systems results in an image noise and radiation dose reduction. We quantify this small pixel effect in measurements of semi-anthropomorphic abdominal phantoms of different sizes as well as in a porcine knuckle in the first clinical PCCT system by using the UHR mode (0.2 mm pixel size at isocenter) in comparison to the standard resolution mode (0.4 mm). At different slice thicknesses (0*.*4 up to 4 mm) and dose levels between 4 and 12 mGy, reconstructions using filtered backprojection were performed to the same target spatial resolution, i.e., same modulation transfer function, using both detector modes. Image noise and the resulting potential dose reduction was quantified as a figure of merit.

**Results:**

Images acquired using the UHR mode yield lower noise in comparison to acquisitions using standard pixels at the same resolution and noise level. This holds for sharper convolution kernels at the spatial resolution limit of the standard mode, e.g., up to a factor 3.2 in noise reduction and a resulting potential dose reduction of up to almost 90%.

**Conclusion:**

Using sharper convolution kernels, UHR acquisitions allow for a significant dose reduction compared to acquisitions using the standard detector mode.

**Clinical relevance:**

Acquisitions should always be performed using the ultra-high resolution detector mode, if possible, to benefit from the intrinsic noise and dose reduction.

**Key Points:**

• *Ionizing radiation used in computed tomography examinations is a concern to public health.*

• *The ultra-high resolution of novel photon-counting systems can be invested towards a noise and dose reduction if only a spatial resolution below the resolution limit of the detector is desired*.

• *Acquisitions should always be performed in ultra-high resolution mode, if possible, to benefit from an intrinsic dose reduction.*

**Supplementary information:**

The online version contains supplementary material available at 10.1007/s00330-023-10499-1.

## Introduction

X-ray-based medical imaging techniques expose patients to ionizing radiation which remains a concern to public health. There is a growing interest in reducing radiation doses while maintaining diagnostic image quality. One emerging technology in the field of computed tomography (CT) is photon-counting CT (PCCT). PCCT offers many advantages over conventional energy-integrating CT (EICT). The detectors employed in EICTs use a scintillator to convert incoming X-ray photons into visible light that is consequently detected by a photodiode and converted into electric signals [1, 2]. In contrast, photon-counting (PC) detectors are able to distinguish photons individually. They employ a semiconductor material wherein the incoming X-ray photons are directly converted into a measurable charge cloud that is transported to electrodes or pixels by an applied bias voltage. The electric pulse generated by this is in the order of tens of nanoseconds [3, 4] and is short enough such that single X-ray photons can be counted individually [5]. To be able to achieve this, not only high-speed electronics but also very small pixel sizes are required to, e.g., avoid pile-up effects. The difference between energy-integrating and photon-counting detectors constitutes several advantages of novel PCCT systems over conventional EICT. These benefits include but are not limited to a reduction of administered radiation dose, lower noise levels in general, partially caused by the absence of electronic noise and statistical effects [6, 7], an improvement in iodine contrast [8], and the acquisition of dual- or even multi-energy data. Last but not least, the small size of the detector pixels in PCCTs allows for increased spatial resolution compared to conventional systems. Although the high spatial resolution may not always be required in clinical practice, theoretical studies have shown that data acquired in the high-resolution modes of PCCT detectors yields better image quality in terms of lower image noise when reconstructed below the resolution limit of the system [9, 10]. This was also verified experimentally in early prototypes of PCCT systems and the results illustrated that a lower noise level can indeed be realized for data acquired with the high-resolution detector modes when the desired modulation transfer function (MTF) is below the resolution limit of the system [11–14]. This effect will be referred to as the small pixel effect in the following. Whereas these studies were performed using preclinical prototypes, the dual-source CT system Naeotom Alpha (Siemens Healthineers) is the first available clinical photon-counting CT system that is capable of harnessing this effect. Hence, we herein aim at evaluating the small pixel effect in this clinical system and at quantifying the potential dose reduction it might offer in clinical practice.

## Materials and methods

### Theoretical foundations

Theoretical considerations and results obtained using prototype systems have illustrated that detector pixel binning should always be avoided [9–11, 14, 15]. Acquisitions using unbinned detectors allow for an image noise and radiation dose reduction compared to acquisitions with larger pixels when reconstructed to the same spatial resolution [9, 10]. This effect can be explained by considering two systems, A and B, with small and large detector pixels. Moreover, let the acquisition geometry of both systems be the same. System A with small detector pixels will achieve a higher spatial resolution than system B with larger detector pixels. Consequently, at the same dose level, the image noise level of system A will be higher than that of system B, when data are reconstructed to the resolution maximum of each system. However, if system A is reconstructed to the resolution of system B, the noise of system A drops below that of system B, namely* σ*_A_ < *σ*_B_, at the same dose level since

1 $${\sigma }^{2}\propto \int \frac{{{\text{MTF}}\left(f\right)}^{2}}{{S\left(f\right)}^{2}}df$$

holds. Therein, MTF(*f*) is the modulation transfer function the data are reconstructed to and* S*(*f*) is the pixel presampling function. It can be shown that the relation *S*_A_(*f*) > *S*_B_(*f*) holds; i.e., smaller detector pixels provide a larger presampling function in frequency domain, and hence image noise is lower for such detectors at the same dose and resolution. A derivation of Eq. (1) can be found in the literature [9, 10].

### CT system

The first clinically available photon-counting CT system (Naeotom Alpha, Siemens Healthineers, see supplemental Fig. 1) was used for all acquisitions in this study. This dual-source system provides a field of measurement (FOM) of 50 cm for the first source-detector-thread whereas the second thread only provides a FOM of 36 cm [16]. Each of the two photon-counting detectors can be operated in two distinct modes: In case of the ultra-high resolution (UHR) mode, the detector provides a pixel size of 151 × 176 µm^2^ in the center of rotation allowing for a spatial resolution of up to 42.9 lp/cm (MTF_10%_) with a collimation of 120 × 0.2 mm at the isocenter [17]. The second mode, herein referred to as standard (Std), results from a 2 × 2-binning of UHR-pixels providing a pixel size of 302 × 352 µm^2^ at the isocenter and a collimation of 144 × 0.4 mm [17]. To maximize the spatial resolution for each detector mode, the X-ray source is operated with different focal spot sizes. In case of Std, the tube provides a focal spot size of 0.8 mm. In case of the UHR mode, the tube provides a focal spot size of 0.4 mm.

### Phantoms and image acquisition

To evaluate the noise and dose reduction of UHR over Std, a semi-anthropomorphic abdomen phantom (QRM, A PTW Company) is used. This phantom can be equipped with fat extension rings to mimic different patient sizes, namely S (20 × 30 cm), M (25 × 35 cm), and L (30 × 40 cm, see supplemental Fig. 1 right). Dose-matched acquisitions are performed using UHR and Std modes at dose levels of 4 mGy, 8 mGy, and 12 mGy (CTDI_32cm_) to cover a wide range of clinically relevant dose levels. Different radiation doses were obtained by adjusting the effective tube current time product, at a tube voltage of 120 kV. Tube current modulation was disabled in all measurements to limit the influence of confounding factors. Image reconstruction was performed with weighted filtered backprojection (wFBP) onto a matrix of 1024 × 1024 pixels with a pixel size of 0*.*293 mm and varying slice thicknesses between 0*.*4 and 4.0 mm. Note that the pixel size was chosen to account for the high frequencies obtained in reconstructions using sharp kernels. Reconstructions were obtained using all available body-regular kernels Brxy whereas lower values of “xy” indicate smoother kernels and higher numbers indicate sharper kernels.

### Noise and potential dose reduction

**Fig. 1:**
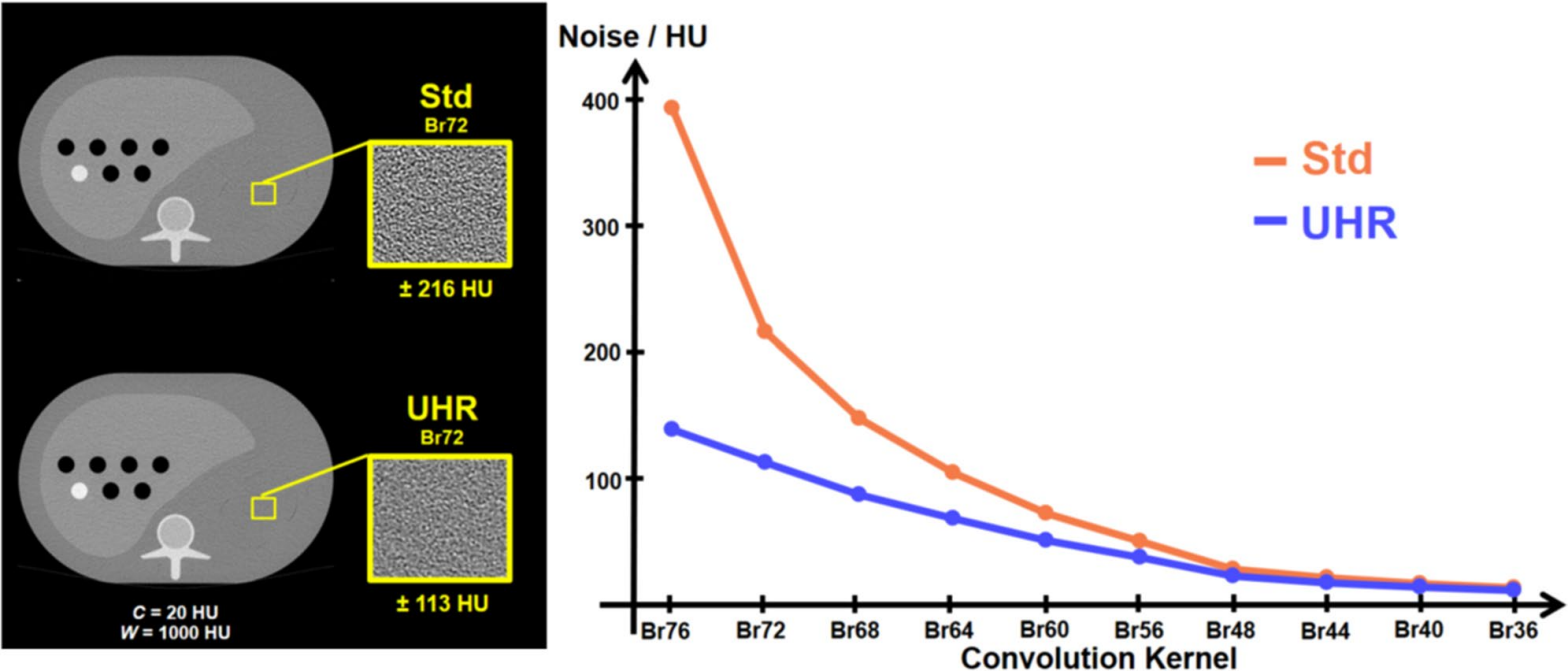
Acquisitions using Std (top left) and UHR (bottom left) detector modes at a dose of 8 mGy reconstructed using a Br72 kernel and a slice thickness of 1 mm.* C* = 20 HU,* W* = 1000 HU. Image noise (in HU) as function of convolution kernels (right)

Image noise was evaluated as the standard deviation of CT values in a homogeneous region of interest (ROI) in difference images after correction for the increase in noise due to the subtraction. In all experiments, this ROI was placed in the area of the phantom’s spleen. This ROI is shown in a regular slice in Fig. 1 for illustration purposes. The image noise* σ*_UHR_ and* σ*_Std_ in reconstructions obtained using the UHR and Std mode, respectively, is measured in several slices of the z-invariant phantom. The ratio *R* of these noise values, i.e.,

2 $$R=\frac{{\sigma }_{{\text{Std}}}}{{\sigma }_{{\text{UHR}}}}$$

is a direct quantification of the small pixel effect and is evaluated at every convolution kernel. The potential dose reduction D^*^ can be derived as.

3 $${D}^{*}=1-\frac{1}{{R}^{2}}=1-\frac{{\sigma }_{{\text{UHR}}}^{2}}{{\sigma }_{{\text{STD}}}^{2}}$$

Furthermore, edge spread functions (ESFs) are evaluated to verify that the images obtained using UHR and Std acquisition modes provide similar MTF(*f*), i.e., a similar spatial resolution, when reconstructed using the same convolution kernels. In particular, edges perpendicular to a linear section of the phantom between air and the homogeneous phantom background are measured in reconstructions with a slice thickness of 0.40 mm and using all available kernels. To account for image noise, 50 edges are averaged per kernel. In a consequent step, the derivative of the ESF was computed to obtain the line spread function (LSF). The Fourier transform of the LSF yields the modulation transfer function (MTF) as measure of spatial resolution. This evaluation was performed using commercial software (RayConStruct-IQ, RayConStruct).

## Results

### Spatial resolution

A prerequisite for the comparison of reconstructions obtained using the UHR and Std modes is the fact that the same reconstruction kernels result in the same spatial resolution, no matter the used detector mode. This was verified for all used kernels by measuring the modulation transfer function (MTF) between air and the homogeneous soft tissue background. Such an MTF is exemplarily shown for a Br40 kernel, i.e., a rather smooth body kernel, using a dose level of 8 mGy (see supplemental Fig. 2). As expected, the figure indicates the same spatial resolution in both reconstructions. This finding approximately holds for all investigated dose levels and reconstruction kernels.

### Results for a slice thickness of 1 mm

In case of a slice thickness of 1 mm, image noise in UHR and Std reconstructions is similar for smoother reconstruction kernels. In case of sharper convolution kernels, the ratio* R* increases monotonically when approaching the resolution limit of the Std pixels. This is shown in an exemplary reconstruction of the S-phantom using a dose of 8 mGy in Fig. 1 for a Br72 kernel. Using Std results in a noise of about 216 HU whereas an acquisition using UHR results in a noise level of 113 HU at the same dose. Additionally, the noise comparison at each available kernel for the dose level of 8 mGy is also presented in Fig. 1. It is obvious that for a low spatial resolution, both acquisition modes yield similar noise levels. However, as the sharpness of the kernels increases, the curves differ significantly. In this particular example, this difference becomes apparent starting at a Br56 kernel. At the highest spatial resolution, i.e., the Br76 kernel, the noise ratio achieves a value of* R* = 2*.*84 which translates into a potential dose reduction  D^*^ of about 87*.*6%.

As a function of dose and phantom size,* R*-values up to a range of 0*.*88 to 3*.*18 can be observed over all phantoms. The maximal potential dose reductions for the M-phantom and the L-phantom are about 88% and just over 90%, respectively. The latter being a measurement with a dose level of 4 mGy. Altogether, the noise level increases with larger phantom size due to an increase in intersection lengths. Consequently, the noise ratio* R* decreases with increasing phantom size. Moreover, the standard deviation of R is higher at a lower spatial resolution, i.e., smoother convolution kernels, and decreases as the spatial resolution increases.

**Fig. 2:**
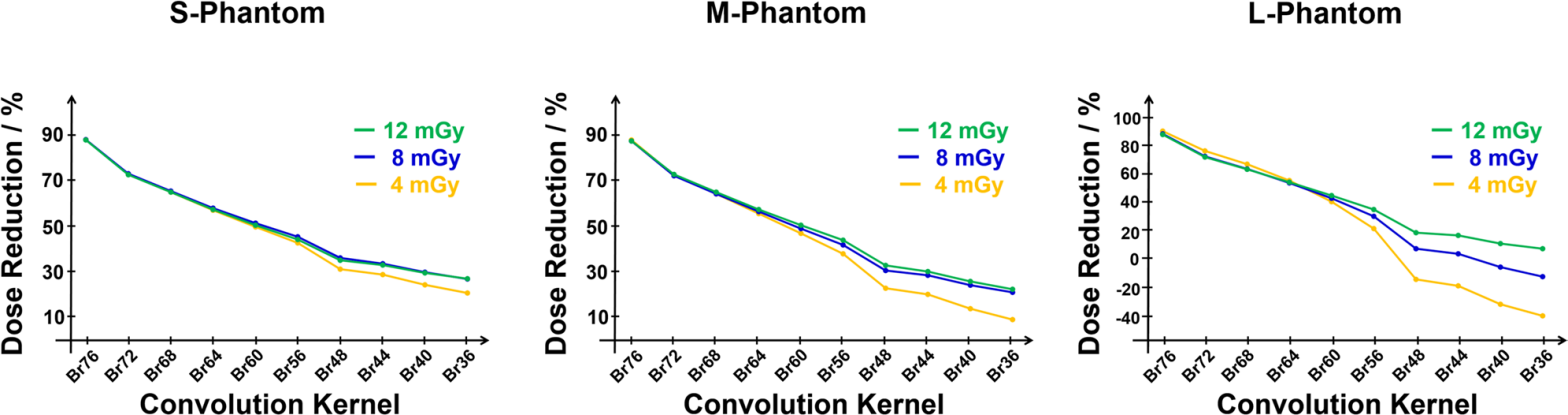
Potential dose reduction* D*.^*^ as function of convolution kernels for the small (left), medium (middle), and large (right) phantom. Each plot illustrates the results obtained using dose levels of 4 mGy (yellow), 8 mGy (blue), and 12 mGy (green)

Clinically, most relevant is the potential dose reduction of UHR over Std. A summary of dose reduction as a function of the phantom sizes and all investigated dose levels is presented in Fig. 2. Each plot represents a different phantom size and contains three different dose reduction curves for the corresponding three dose levels of 4 mGy, 8 mGy, and 12 mGy. In the particular case of smooth kernels, the dose reduction achieved at different dose levels at a given phantom size diverges. This effect is particularly apparent in case of the L-phantom. However, in all cases, the dose reduction curves converge at the sharpest kernel.

### Influence of slice thickness

**Fig. 3:**
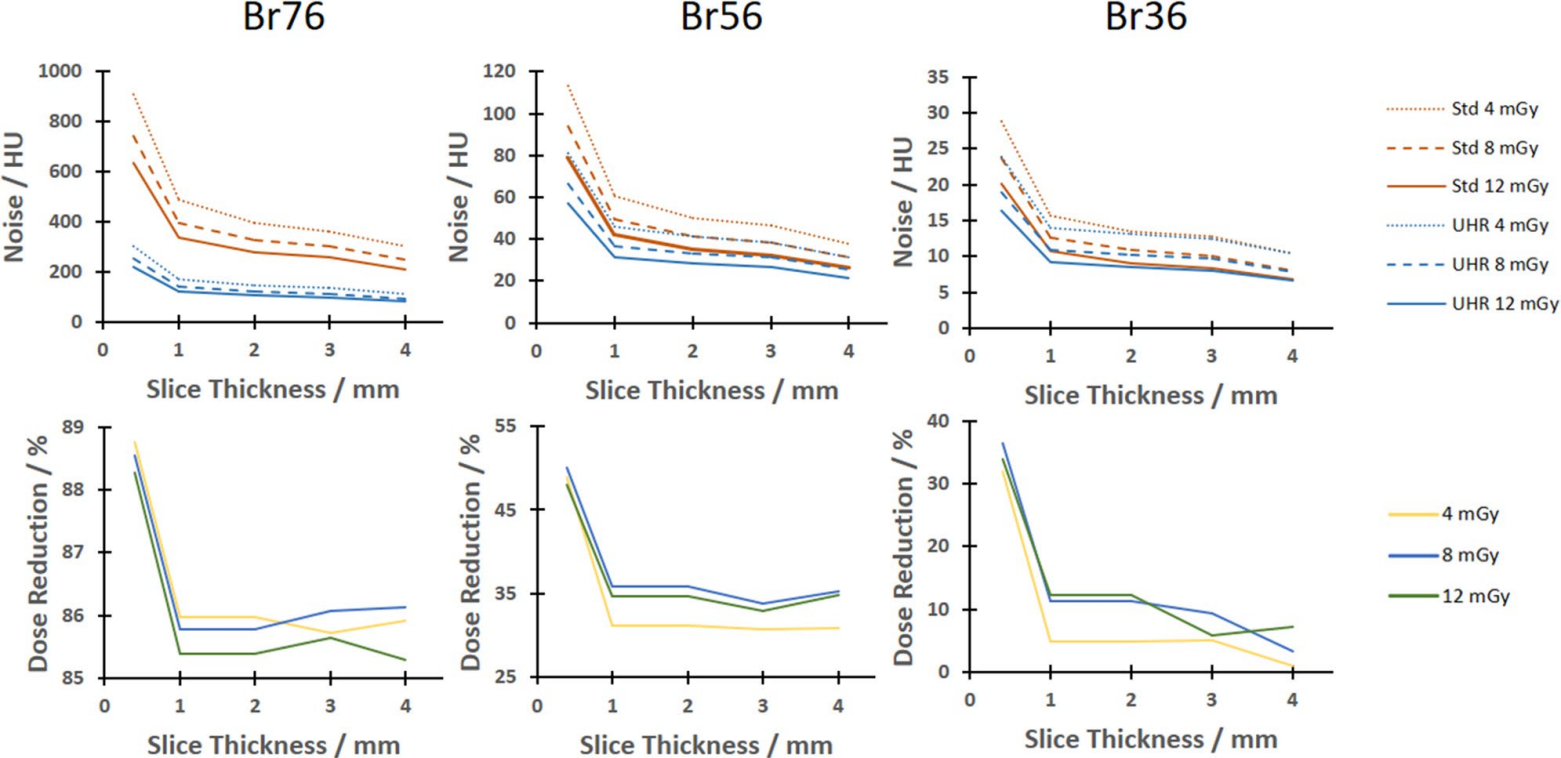
Image noise as function of slice thickness for the Br76 kernel (top left), the Br56 kernel (top middle), and the Br36 kernel (top right) for all investigated dose levels in the S-phantom. Similarly, the bottom row shows the potential dose reduction for these scenarios

Usually, abdominal scans are reconstructed at a slice thickness of about 2 to 5 mm [11], yet, theory states that the advantage of acquisitions using a small pixel size decreases if thick slices are reconstructed [9]. This dependence on slice thickness is exemplarily illustrated in Fig. 3 using the S-phantom for a very sharp Br76 kernel (left), a sharp Br56 kernel (center), and a smooth Br36 kernel (right). The first row of that figure shows image noise for the UHR and Std acquisitions at all dose levels. For the sharpest kernel, a clear difference between the two acquisition modes can be seen. Moreover, thinner slice thicknesses yield higher noise levels in general yet the noise ratio* R* is larger. Figure 3 shows the potential dose reduction, illustrating that D^*^ is maximized at sharper kernels, especially at a slice thickness under 1 mm.

**Fig. 4:**
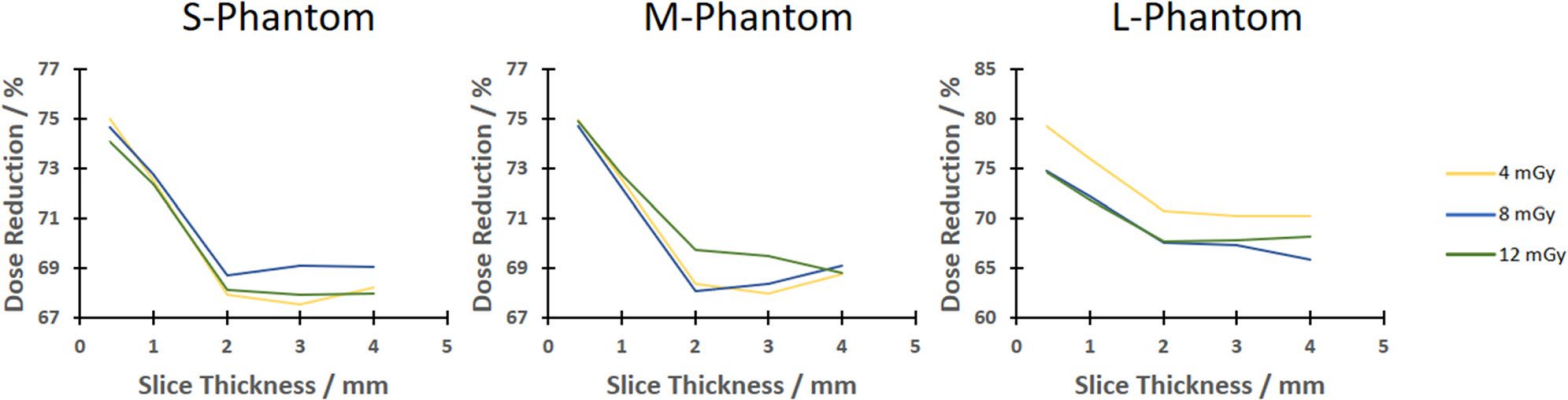
Potential dose reduction as function of slice thickness for the Br72 kernel for the S-phantom (left), M-phantom (middle), and L-phantom (right)

Since Fig. 3 only provides results obtained using the S-phantom, Fig. 4 illustrates the dependence on slice thickness for all phantom sizes using a Br72 kernel; i.e., the plot shows the potential dose reduction obtained using the S-phantom (left), M-phantom (middle), and L-phantom (right). It can be noticed that for a slice thickness of 2 mm and larger, the potential dose reduction is very similar over all dose values. However, for a slice thickness of 0*.*4 mm, which is also the minimum for the Std acquisition mode, a slight increase of dose reduction is apparent. In case of higher slice thicknesses, a dose reduction of at least 69% was reached for all three phantom sizes. All in all, these results further highlight the benefit of acquisitions in an unbinned detector mode and additionally demonstrate an advantage at reconstructions with a slice thickness below 2 mm.

### Practical example

**Fig. 5:**
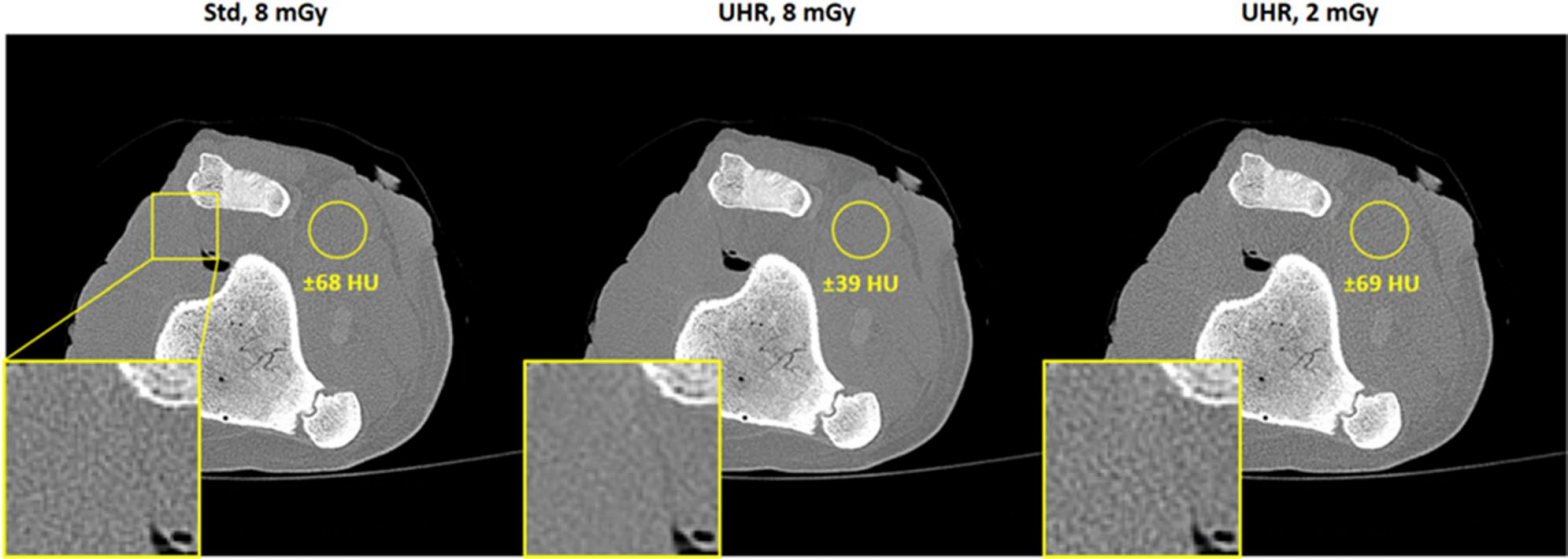
Images of a porcine knuckle acquired with the Std (left) and the UHR mode (middle) at 8 mGy reconstructed with a Br72 kernel. Image noise in UHR acquisitions was further reduced to match the noise found in the Std acquisition and resulted in a dose of 2 mGy (right). *C* = 575 HU,* W* = 2600 HU

To further illustrate the difference between UHR and Std, a porcine knuckle was examined at a dose of 8 mGy (CTDI_32cm_) and a slice thickness of 1 mm. Reconstructions were performed using a Br72 kernel, i.e., a kernel for bone imaging. Furthermore, acquisitions with reduced tube current have been performed in UHR mode to match the noise found in the standard mode acquisition conducted at 8 mGy. Figure 5 illustrates the cross-sections of the sample. Image noise is measured in the same circular ROI and magnifications show soft tissue as well as cortical and cancellous bone. Image noise in case of acquisitions using 8 mGy show noise levels of 68 HU and 39 HU for Std and UHR mode, respectively (Fig. 5 left and middle). This corresponds to a noise reduction of about 43% and a theoretical dose reduction of about 67%. To experimentally verify this dose reduction, the tube current in UHR acquisitions was reduced to approximately match the noise in acquisitions using the standard mode (Fig. 5 right). It was found that this is achieved at a dose of 2 mGy, resulting in a noise level of 69 HU in the corresponding UHR acquisition compared to 68 HU found in the standard acquisition.

## Discussion

Herein, the effect of noise and potential dose reduction because of the small pixel effect in the first clinically available PCCT was assessed. Acquisitions using the binned (Std) and the unbinned (UHR) detector modes were compared in phantom measurements and in a porcine knuckle. The noise level in UHR acquisitions was generally lower compared to Std acquisitions if compared at the same MTF and dose. This effect was particularly prominent when using sharp convolution kernels. This is in accordance with theoretical expectations and simulations [9, 10] and results obtained using an experimental system [11]. The noise ratio* R* measured using the two detector modes reached values of up to 3*.*18 for sharpest kernels translating to a potential dose reduction of more than 87% at a slice thickness of 1 mm. Reducing the slice thickness to 0*.*4 mm increased the potential dose reduction up to almost 89% for sharp kernels outperforming previous studies in research systems [11–13, 18]. This is most likely caused by a combination of overall smaller detector pixels and a smaller focus size of the clinical system.

Using smoother reconstruction kernels resulted in an overall reduction of the potential dose reduction of UHR over Std. For the smoothest kernel, no benefit of UHR over Std was found which corresponds to theory [19]. Interestingly, the potential dose reduction did show a dependence on both phantom size and administered radiation dose (see Fig. 2). This rather unexpected behavior might be explained by the intrinsic application of signal-dependent filters that operate on the acquired raw data as a function of administered dose and detector mode. Furthermore, the application of moiré filters might limit the achievable spatial resolution of the system and explain the convergence of potential dose reductions at sharper kernels. A slice thickness of 0*.*4 mm showed the highest potential dose reduction among all investigated slice thicknesses. This could be explained by the fact that for slice thicknesses of 1 mm and above, which are also larger than the resolution limit in the isocenter, the small pixel effect in longitudinal direction is reduced and the results are governed by the small pixel effect in axial direction. However, at thinner slices, both effects would mix. This gains particular relevance when following the well-known paradigm of “reconstruct thin, view thick.”

**Fig. 6:**
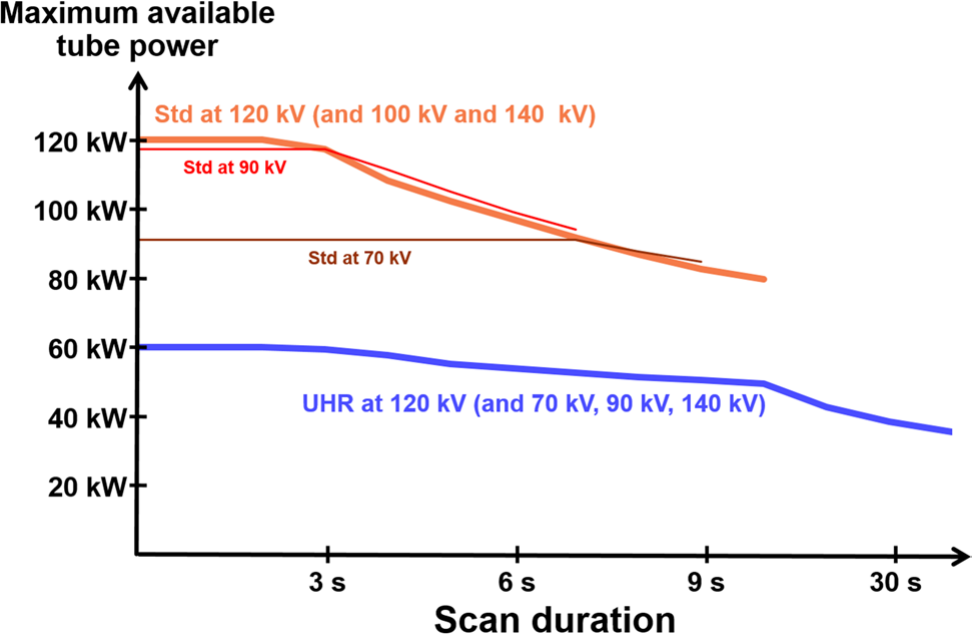
Maximum tube power for UHR and Std acquisitions. The Std mode achieves the maximal available tube power of 120 kW for the Vectron X-ray tube at tube voltages of 100 kV, 120 kV, and 140 kV. The UHR mode, however, can only be operated at lower tube powers. Adapted from Sartoretti et al [20]

Similar results were obtained in a porcine knuckle (see Fig. 5). At the used high spatial resolution, the noise level of UHR is far lower compared to Std, resulting in a ratio of* R* = 1.7 and a potential dose reduction of just over 67%. The results presented herein indicate that in clinical practice, the UHR mode along with thin slices and sharp convolution kernels should be the standard if possible. However, there are some drawbacks to the usage of the UHR mode. A major challenge that arises in UHR mode is a physical one. The achievable tube power in UHR mode is only half of the achievable power when using the Std mode when operating at 120 kV. This is illustrated in Fig. 6 wherein the maximum tube power is shown as function of scan duration for both modes at all available tube voltages. Since the UHR mode employs a smaller focal spot, the available tube power has to be reduced to protect the anode [19]. This, for example, may prohibit UHR measurements in obese patients and may prohibit the use of patient-specific tin prefiltration. Data for this plot were generated by continuously increasing the tube current for a given protocol and tube voltage at the scanner’s control console until scanning was no longer possible indicated by a corresponding error message.

This study, however, encounters some limitations. Firstly, this investigation has not been performed using patients, only measurements with phantoms and a porcine knuckle were performed. Secondly, neither did we investigate the influence of tube voltage nor the iterative reconstruction algorithms on the small pixel effect. Tube voltage should barely affect it and in this study it was actively decided to only reconstruct using FBP, as the non-linearity in iterative reconstruction algorithms may alter the MTF. However, it may be still interesting to address the latter in future studies [20]. Furthermore, the exclusive consideration of physical quantities, such as noise magnitude and spatial resolution, only partially mimics clinical reality. Tasks such as the detection of low contrast lesions are, for example, dependent on the actual noise texture [21]. Additional studies are required that investigate the potential effect of the dose reduction reported herein on specific clinical tasks as has been presented for lung imaging recently [22].

In summary, the experiments presented herein show that the utilization of the UHR scanning mode should be used, along with thin slice reconstruction, as often as possible in the first clinical PCCT. Bone imaging will especially profit, as sharper kernels profit greater from the small pixel effect. This applies in particular if high spatial resolution is not required and either low noise levels or a low administered radiation dose is vital for the diagnostic task at hand.

### Supplementary information

Below is the link to the electronic supplementary material.

Supplementary file1 (PDF 160 KB)

## Abbreviations

CT: Computed tomography
CTDI: CT dose index
EICT: Energy-integrating CT
ESF: Edge spread function
FOM: Field of measurement
LSF: Line spread function
MTF: Modulation transfer function
PCCT: Photon-counting CT
ROI: Region of interest
Std: Standard detector mode
UHR: Ultra-high resolution
wFBP: Weighted filtered backprojection

## Declarations

### Guarantor

The scientific guarantor of this publication is Dr. Stefan Sawall.

### Statistics and biometry

No complex statistical methods were necessary for this paper.

### Study subjects or cohorts overlap

No study subjects or cohort overlap reported.

### Methodology

• Experimental

• Performed at one institution
