# Supplementary material for: Potential radiation dose reduction in clinical photon-counting CT by the small pixel effect: ultra-high resolution (UHR) acquisitions reconstructed to standard resolution
Source: Eur Radiol. 2023 Dec 22;34(7):4484–91. doi: 10.1007/s00330-023-10499-1 (PMC11213748; doi:10.1007/s00330-023-10499-1)

**Suppl Figure 1:** On the left side, the Naeotom Alpha photon-counting CT system. On the right, the semi-anthropomorphic abdomen phantom and the fat extension rings used in this study are shown**.**

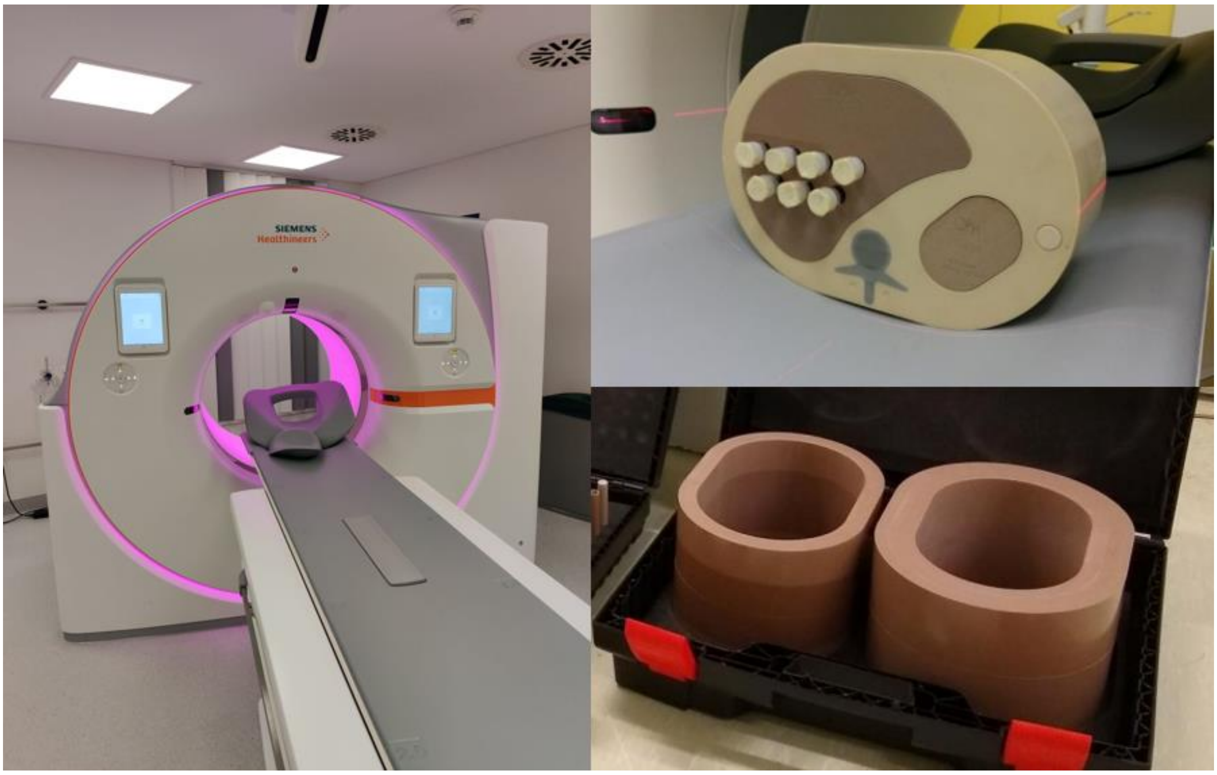

**Suppl Figure 2:** Modulation transfer function (MTF) between air and the homogeneous soft tissue background of the phantom for the UHR (green) and Std (purple) acquisition mode reconstructed using a B40 kernel at a slice thickness of 1 mm and a dose of 8 mGy. On the left, the corresponding profiles in an S-phantom reconstruction. *C* = 20 HU, *W* = 1000 HU.

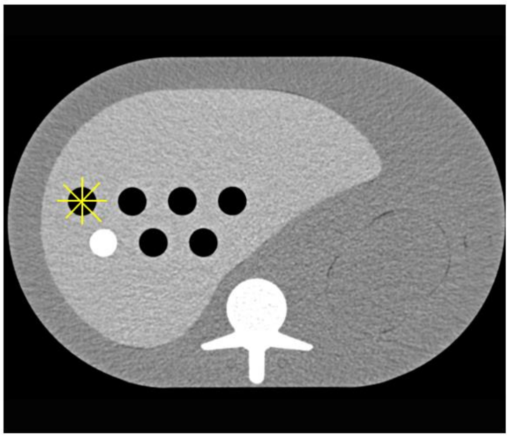

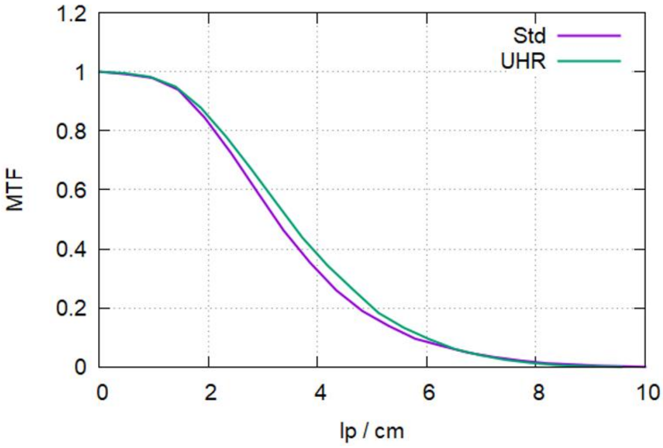

Supplement: Supplementary file 1 — Supplementary file1 (PDF 160 KB) [file 330_2023_10499_MOESM1_ESM.pdf]
